# Multilevel factors influencing eHealth adoption among older adults during the pandemic

**DOI:** 10.3389/fpubh.2025.1531173

**Published:** 2025-04-28

**Authors:** BoRin Kim, Chung Hyeon Jeong, Emily Blood, Sajay Arthanat, Marguerite Corvini, Casey Golomski, John Wilcox

**Affiliations:** ^1^Department of Social Work, University of New Hampshire, Durham, NH, United States; ^2^Department of Occupational Therapy, University of New Hampshire, Durham, NH, United States; ^3^Center for Digital Health Innovation, University of New Hampshire, Durham, NH, United States; ^4^Department of Anthropology, University of New Hampshire, Durham, NH, United States

**Keywords:** ehealth adoption, qualitative research, multilevel, push factors, pull factors, mooring factors

## Abstract

**Introduction:**

While eHealth became prevalent in healthcare during the pandemic, eHealth adoption by older adults has been slow. This digital divide could lead to worsening health and healthcare disparities. Guided by the push-pull-mooring perspective, this study explored factors influencing eHealth adoption among older adults.

**Methods:**

Semi-structured virtual interviews were conducted in 2021 with 31 older adults with healthcare needs. Interviews were thematically coded and analyzed using constant comparative methods.

**Results:**

We found the pandemic to be a powerful push factor that forced older adults to adopt eHealth along with several pull factors that attracted older adults to use eHealth. A number of mooring factors that made older adults hesitant to adopt eHealth were identified: limited knowledge or skills on eHealth, security concerns, limited technology access, and peer influence.

**Discussions:**

Our findings indicate areas to increase productive use of eHealth within older populations, such as education or support within social networks.

## Introduction

eHealth refers to the cost-effective and secure use of information and communication technologies (ICT) in support of health-related fields, which includes telehealth, mobile health (mHealth), electronic medical or health records (eMR/eHR), and wearables (57). During the COVID-19 pandemic, eHealth rapidly became a major force in the effort to provide high-quality service in the safest possible environment (1). The effectiveness of eHealth has been promising for many health areas including chronic condition care and management, health promotion, and behavioral risk reduction (2, 3, 40, 42, 48–50, 53, 54). Health service providers have attempted to get necessary equipment to people in need and to teach their clients how to use these platforms (4).

Despite the great needs for eHealth among older adults, older adults tend to be digitally disadvantaged, and they have been slow to embrace eHealth as an alternative to traditional in-person medical care (5). Since the beginning of the pandemic, eHealth has been instrumental for both providers and patients, but this digital divide may lead to increasing health and healthcare disparities (43, 51, 52). Therefore, there is an urgent need for developing policies and programs that can support older adults to adopt eHealth for their health needs (45).

The present study aims to contribute to the current knowledge of eHealth readiness among older adults by exploring the factors that positively and negatively influence older adults’ intentions to adopt eHealth. Two theoretical frameworks allowed us to investigate factors associated with eHealth adoption among older adults more comprehensively and elaborately. First, a push-pull-mooring (PPM) framework guided us to investigate various factors that force older adults to adopt eHealth (i.e., push factors) or attract older adults to adopt eHealth (i.e., pull factors), or make them hesitant to adopt eHealth (i.e., mooring factors). Second, the theory of acceptance and use of technology (UTAUT) was used to identify multilevel factors including individual-, interpersonal-, and environmental-levels that could lead to eHealth adoption.

### The push-pull-mooring perspective

The PPM perspective was used as a conceptual framework for identifying facilitators of and barriers to eHealth adoption among older adults. The PPM was first proposed to understand human migration decisions (59). The PPM indicates how human behavior is affected by push, pull, and mooring factors. Push factors drive people away from their place of origin. Pull factors attract people to a new place, and mooring factors constrain people’s movement. More recently, the PPM perspective has been used widely in various areas of research focusing on changes in decision-making. Several studies have shown that the PPM is a useful framework for investigating human decisions to make changes such as the use of mobile services (58), telelearning (6), and mobile medical platforms (60).

Switching from traditional in-person doctor visits to eHealth can be a big decision for many patients, particularly for older adults who have established strong, in- and inter-personal relationships with healthcare providers over a lifetime and have relatively limited experience with ICTs. They tend not to be knowledgeable about or have limited skills in using ICTs. As mentioned above, there have been few studies focusing on older adults’ intention to adopt eHealth. Some studies have identified barriers to telehealth or other Internet-based healthcare service use among older adults which included: lack of desire, self-confidence, technical literacy, technology access, low privacy or security, low trust, and high switching costs (7, 8, 61). Regarding facilitators of eHealth, Lai and Wang (61) found that personalized care, ubiquitous care, and responsiveness influenced older Taiwanese’s intentions to switch to cloud healthcare services. There is still limited knowledge on what factors positively influence older adults’ intentions to adopt eHealth. The PPM framework could allow us to explore various types of factors that affect the decision to switch to eHealth. These findings would be helpful in the development of effective programs and services for older adults’ eHealth adoption.

### The unified theory of acceptance and use of technology

Guided by the UTAUT (9), this study identified multi-level factors that influence eHealth adoption among older adults. The UTAUT is the most influential user acceptance and behavior analysis theory and has been widely used to explain intention to adopt a range of ICTs. Performance expectancy, defined as the degree to which an individual believes that using the technology will help them to achieve gains in job performance, reflects the perceived usefulness and relative advantage associated with using ICTs. Effort expectancy, which refers to the degree of ease associated with the use of the system, is associated with perceived ease of use. If users need to invest great effort in learning to use or skillfully use ICTs, they may not adopt or discontinue use. Social influence reflects the degree to which an individual perceives that someone important to them believes they should use the new system. When others who are valued by a user recommend that they use ICTs, they may follow their suggestions. Lastly, facilitating conditions reflect the degree to which an individual believes that an organizational and technical infrastructure exists to support the use of the system. Resources such as Internet connection, ICT devices and perceived financial barriers are considered as facilitating conditions. A number of studies from various fields have demonstrated that the UTAUT is a comprehensive and reliable theory that can explain as much as 70% of the variance in intention (9, 10). Examining the presence of each of these constructs allows for identifying key influences on acceptance of technology (11).

Although an emerging line of research has applied the UTAUT in healthcare research, most healthcare research using the UTAUT has focused on attitude and adoption of ICTs among hospitals and healthcare providers (12, 13). In recent years, an increased number of studies have focused on patients’ use of ICTs with the framework of the UTAUT. These studies explored various areas in eHealth including telehealth (14) mHealth (15, 16), and an electronic health record patient portal (17). These studies have demonstrated that the four core constructs addressed from the UTAUT are significant factors influencing patients’ attitudes toward and adoption of eHealth. As discussed above, the few studies of older patients’ eHealth adoption focused on barriers or individual-level factors only.

To fill the gaps in knowledge, this study explored push, pull, and mooring factors influencing eHealth adoption among older adults, with a multi-level lens including individual-level, interpersonal-, and environmental-level factors through in-depth interviews with older adults from different socioeconomic and residential backgrounds,

## Methods

### Participants

We recruited 31 older adult (age 60+) participants from various socioeconomic classes, including those living in subsidized senior housing, Continuing Care Retirement Communities (CCRC), 55+ communities, and their own homes in regular communities. The sample size was determined based on literature on pretesting qualitative research methods (18–20), which recommend a sample size of around 30 for data saturation. We utilized a purposive sampling strategy for a well-informed selection of very specific cases, capable of maximizing the chances of observing phenomena of interest, which is suitable for qualitative studies with a small sample size (21). The sample inclusion criteria were: (a) living independently without any help from caregiver, (b) living in the areas covered by any broadband services, and (c) having at least one chronic condition and/or ongoing health issues. Participants were excluded if they had a cognitive impairment or a progressive disorder which could impact the interview. All study procedures were approved by the Institutional Review Board (IRB) of the University (#8448 blinded for review).

To recruit participants, we reached out to several community partners working with older adults in Northern New England via phone or email. These included library directors, service coordinators of subsidized senior housing communities and CCRCs, representatives of groups of older adults, and the associations of senior centers around the area. Our community partners connected us with potential research participants who met our inclusion and exclusion criteria. They also helped to distribute our fliers and to advertise our study through their newsletters.

### Data collection

Data collection took place between June and September 2021. Individuals who revealed intent to participate in the study contacted our research team through email or phone, and we conducted a telephone screening interview to determine eligibility for participation. If a participant met our inclusion and exclusion criteria, we scheduled an interview with them. An interview packet with an information sheet and consent form was mailed to the participants one week before the scheduled interview. A total of 31 semi-structured interviews were conducted remotely via video conference (e.g., Zoom) from the participants’ location, with a phone interview option offered as well. If a participant lacked a device for Zoom or access to Wi-Fi, the research team provided them with an iPad and/or a wireless router that served as a mobile Wi-Fi hotspot (e.g., MiFi) to facilitate their participation. If participants needed assistance in utilizing the video conference tools, our research team offered support to set up and participate in the online interview through phone or in-person visit. Due to COVID-19, in-person contacts were minimized to prevent potential disease transmission. Nonetheless, if an in-person visit was needed to help set up a Zoom interview, the research team adhered to the CDC guidelines to ensure the safety of both study participants and research team members (i.e., meeting outside, wearing masks, maintaining at least six feet of distance, hand sanitizing before the meeting). Only six participants needed some assistance, and they all lived in subsidized senior housing communities.

At the beginning of the interview, interviewers confirmed the interviewees’ intent of participation. To ensure a consistent understanding of eHealth among all participants, each interview began with a clear and comprehensive definition of eHealth (i.e., telehealth, patient portals, mHealth, and any internet or mobile applications used for healthcare), along with specific examples. The main interview included a series of questions in the following areas, guided by UTAUT: personal experiences or knowledge of eHealth, personal skills on using eHealth, and potential risk/protective factors of eHealth readiness both at an interpersonal level (e.g., social support from family members or neighbors) and at an environmental level (e.g., public computer availability, computer education programs at senior housing). All the interviews were recorded with Zoom under the interviewees’ agreement. Participants received a $30 gift card by mail or email after completing interviews.

### Data analysis

We conducted a thematic analysis using the constant comparison method to identify themes grounded in the interview data. The video/audio recordings from the interviews were first transcribed verbatim and then thematically coded by two independent coders. During the initial coding phase, we utilized open coding to identify initial categories that emerged from the data. Subsequently, we employed axial coding to group similar codes into broader categories, thus facilitating the identification of common themes and patterns within the data. To improve the reliability of the coding process, we utilized the negotiated agreement approach, in which multiple researchers from five different disciplines collaborated to code a transcript, compare codes, and resolve any disagreements through discussion. The goal of this approach was to reach a final version that was agreeable to all researchers involved (22). Discussion among coders was facilitated to attain a consensus in regard to assigning codes to specific themes and redefining/collapsing themes to create conceptually relevant and mutually exclusive themes. A final list of codes included a set of *a priori* themes derived from questions in the interview guide and emergent themes raised by respondents. To protect participant confidentiality, we identified participants by codes rather than by names both in our analyses and reporting of the study results. This approach not only ensured anonymity but also facilitated a more objective analysis of the data. All the themes identified were categorized into push, pull, and mooring factors and individual-, interpersonal, and environmental levels.

## Results

### Sample characteristics

Table 1 shows the characteristics of our sample. The average age was 74.27 years with a range of 60 and 88. The majority of our sample was female (81%), and 45% lived alone. The education level tended to be high, including 3% less than high school, 23% completed high school, and 74% had more than high school education. Household income level was distributed from very low income (less than $25,000) to high income (more than $75,000). The average number of doctor visits last year was 3.93 with a range of 0–16, and 41% of the sample rated their health either fair or poor. Approximately a quarter of the participants reported that they lived more than 10 miles from the doctor’s office.

**Table 1:** Sample characteristics (*N* = 31).

Variables (Range)	Mean (SD) or %	Variables (Range)	Mean (SD) or %
Age (60–88)	74.27 (3.32)	# of doctor visit last year (0–16)	3.93 (3.58)
Female	81%	Self-rated health	
Education		Very poor	0%
Less than high school	3%	Poor	6%
High school	23%	Fair	35%
More than high school	74%	Good	33%
Household income		Very good	26%
<$25 k	22%	Distance to doctor’s office	
$25 k-$35 k	12%	<3 miles	23%
$35 k-$50 k	22%	3-5 miles	13%
$50 k-$75 k	13%	5-10 miles	42%
>$75 k	39%	>10 miles	23%
Living alone	45%		

Thematic analysis was conducted to identify various facilitators, barriers, and situational contexts shaping older adults’ perceptions, experiences, and decisions regarding eHealth usage. Table 2 summarizes the key themes, subthemes, and representative quotes illustrating the push, pull, and mooring factors influencing eHealth adoption among older adults.

**Table 2:** Themes, subthemes, and representative quotes.

Theme	Subtheme	Quotes
Push factors	Pandemic-induced situation: Social distancing	When the whole COVID thing came up, and doctors shut down and all, my internist in OOO, I had an appointment with him, and they wanted to Zoom it.
	Pandemic-induced emotions: fear and anxiety	During COVID nobody knew how to get healthcare. We were afraid to go to emergency rooms, we were afraid to go to our doctor’s offices.
Pull factors	Perceived usefulness	*Convenience:* It’s immediate, I do not have to wait for my appointment with the doctor, I can just go online and find out what’s going on. *Enhanced patient-provider communication:* I was able to write a message directly to my provider on the portal. And I had a written record of what I said, and a written record of what they said, and I love that actually a lot.
	Perceived ease of use	It was hard at the beginning. But with repetition, it’s so easy. It’s just a matter of just getting the hang of it. So, after several days of doing it, all of a sudden it was easy
	Social Influence	I think, with my knee surgery, my daughter-in-law and I got online with, she was great with online for me. I think that’s what piqued my interest doing things online.
Mooring factors	Personal and psychological concerns	*Security issue*: I’m assuming that my Medicare number will be on there […] someone else will get it […] I’m not a fan of it only because of that.
		*Information overload*: I have not registered on the portal, or whatever they call it for the [a health plan], because I do not want to get all their junk emails and I do not want to have a log in and all that kind of stuff
		*Unfamiliarity with technology*: [Clinic name] sent me an email […] and I just have not done it because I’m not sure of myself […] I’m just not brave enough to do it.
		*Physical limitations: I do not type well […] it’s an effort to send my questions to the doctors […] I’d rather pick up the phone.*
	Lack of social influence	I do not know anybody my age, in my circle and including my friends in their 50s, who do (eHealth).
	Limited infrastructure	I think there’s definitely a need for seniors to be able to have access […] to inexpensive equipment […] inexpensive internet because they are all basically living on fixed incomes

## Push factors

Many respondents pointed out that COVID-19 was the major driving force in their eHealth adoption. Older participants in our study had little to no experience or even awareness of eHealth prior to the pandemic. One respondent indicated,

“*The only thing I can say is, I think that COVID-19 impacted us to do telehealth. I don't think that there was really a lot of telehealth prior to COVID-19. I mean, if there was, I was not involved in it or didn't know about it or didn't do it*.” (ID 1010)

The adoption of eHealth triggered by COVID-19 had both extrinsic and intrinsic aspects: Pandemic-induced situation and pandemic-induced emotion.

### Pandemic-induced situation: social distancing

Social distancing that involves maintaining distance in a physical space between individuals was widely adopted by healthcare providers to reduce transmission of the virus (23). This brought significant changes in the way medical services are supplied by healthcare providers and consumed by patients in healthcare settings. The pandemic has made it inevitable for healthcare to be provided through online platforms, despite the initial lack of preparation during its early stages. Healthcare providers are often the driving force behind the adoption of eHealth. Healthcare consumers had no option but to accept the situation in which healthcare providers only provided medical services via online platforms. As one patient stated,

“*When the whole COVID thing came up, and doctors shut down and all, my internist in [blinded for confidentiality], I had an appointment with him, and they wanted to Zoom it*.” (ID 1009)

Changes in healthcare delivery have resulted in changes in patient consumption, even if patients do not prefer these changes. One participant explained that healthcare was pushing her to use the patient portal, saying, “*Yes, they push it*” (ID1013). We found opposite perspectives/reactions to the utilization of eHealth depending on the respondents’ level of knowledge or skills toward information technology, which impacted their intention to adopt eHealth in the future. Respondents who were accustomed to the traditional face-to-face healthcare services found it difficult to use technology, were dissatisfied with the situation as they were forced to change the way they consume healthcare services. They wanted to go back to the old ways once distancing protocols were lifted.

*“I think we'll go back to the old way of doing it. Their doctor or their nurse or whatever. Most of my friends are comfortable doing it, but they're frustrated. I mean, they're not opposed to it but sometimes they get frustrated.”* (ID1027)

On the other hand, respondents who were relatively comfortable using technology were satisfied with the changes in healthcare services delivery due to the pandemic and indicated that they would continue to use eHealth in the future.

*“Oh, if it's a basic thing, then yeah, I wouldn't mind doing it again. It's definitely much easier to do it at your dining room table than to have to schlep to the doctor's office, for something not major. So, yeah, I would do that again.”* (ID1002)

### Pandemic-induced emotions: fear and anxiety

Despite the need for older adults with chronic conditions to receive continued healthcare during the pandemic, they encountered a sense of fear and anxiety of being exposed to the virus when visiting clinics due to its highly infectious nature (41, 46).

*“During COVID nobody knew how to get healthcare. We were afraid to go to emergency rooms, we were afraid to go to our doctor's offices.”* (ID 1025)

As aforementioned, the adoption of eHealth was generally driven by healthcare providers. However, some patients actively requested healthcare providers to provide eHealth services as a way of protecting themselves due to the fear of being exposed to COVID-19.

*“I actually requested it. […] I wasn't going to go in there and risk getting COVID. […] COVID plus old equals death, then you very quickly start thinking about how to protect yourself. So no, I think it was totally just feeling cautious and wanting not to put myself out there any more than I absolutely had to.”* (ID1003)

We found that older patients’ long-term intentions to adopt eHealth can be influenced by their perceived susceptibility. Older adults may be more motivated to use technology for healthcare when they experience pandemic-induced emotions such as fear and anxiety, which stem from a heightened sense of susceptibility. Conversely, when individuals perceive a low susceptibility to COVID-19, their intention to use eHealth in the long term appears to remain low. The respondent above also stated,

*“I wouldn't see it as a big need right now once COVID, now I'm double vaccinated, and I'm quite comfortable going out. I still drive well. So, if you go just by how I am right now, I probably will do all in-person appointments going forward.”* (ID1003)

## Pull factors

### Perceived usefulness

We found intrinsic factors of eHealth that attracted older adults as healthcare consumers to eHealth by constructing positive perceptions or attitudes toward eHealth. Respondents who were aware of the advantages of eHealth indicated that convenience and usefulness were the key motivating features of eHealth, because it enables patients to have immediate and easy access to healthcare services and health information. As one respondent noted, *“It’s immediate, I do not have to wait for my appointment with the doctor, I can just go online and find out what’s going on”* (ID 1008). Along with concerns about transportation given some older adults’ limited mobility or poorer health conditions, the ability for patients to communicate with their doctors without in-person visits appeared attractive to them.

*“I live 15 miles away from my primary care, I'm a widow, and transportation if I was really sick would be difficult. So, the idea that I might be able to communicate with somebody would be helpful.”* (ID 1025)

Respondents also indicated that use of eHealth could facilitate patient-provider communication by enhancing patients’ ability to access medical records and health information. The respondent who experienced the patient portal emphasized that eHealth could provide patients with more initiative and autonomy in doctor-patient relations.

*“In terms of first time I actually did a tele-visit, the portal was my first personal experience, I guess. And it was amazing actually, […] I was able to write a message directly to my provider on the portal. And I had a written record of what I said, and a written record of what they said, and I love that actually a lot.”* (ID1017)

### Perceived ease of use

Perceived ease of use at individual level was cited to be a critical factor in shaping positive attitudes toward eHealth.

*“It was very easy. They sent me email that had a test link that allowed me to test my device to make sure I was able to connect. And then they sent me, right before the appointment, they told me that an hour before the appointment they would send me the link that I was going to use. So, it was very easy. I really do think that almost anyone could do it. So, I think that might be a good way to transition people into thinking, ‘oh this is kind of a cool way of doing things,’ I like that, I felt good about it.”* (ID1018)

Familiarity with technology—often stemming from respondents’ existing knowledge, skills with electronic devices, or experience from previous jobs—contributed to their perception of eHealth as easy to use. As one respondent described the experience of a virtual appointment with his gastroenterologist:

*“It was like, logging into any site, I do all my banking online, so all those things are, it's no different than, you just know where you have to go. […] it's just like any other site that you go to send a message, to whom, and then I just write out what I want […] once you're doing everything else online, it's just another thing online.”* (ID1022)

Even those who felt eHealth was difficult at first reported that they got used to it quickly after using it repeatedly and started to feel comfortable at some point.

*“It was hard at the beginning. But with repetition, it's so easy. It's just a matter of just getting the hang of it. So, after several days of doing it, all of a sudden it was easy.”* (ID1011)

### Social influence

At the interpersonal-level, we found the pivotal roles of social influence in promoting the adoption of eHealth among older adults. The social influence manifested in various forms, including encouragement or motivation by close social networks as well as the broader social climate for eHealth.

*“Definitely, the peer business, if we can go back to that, is definitely a force that naturally makes me want to use technology more when it comes to health. I think there is a natural, even if it's not my immediate peer group, like there is with MyFitnessPal, there is a national movement towards using eHealth and I want to be part of that movement, I want to be youthful appearing, even though I may not be, but to my own children, and my female friends, and colleagues, that I know what's up.”* (ID 1011)

Social learning through technical assistance provided by individuals in one’s proximate social networks was also identified as a critical factor. It is noteworthy that even older adults who were not technologically adept could benefit significantly from tangible assistance offered by their social networks in using technology for healthcare.

*“I think, with my knee surgery, my daughter-in-law and I got online with, she was great with online for me. I think that's what piqued my interest doing things online because we did the virtual operation by picking and doing, but then we really watched a real one.”* (ID1031)

This exemplifies how social learning can be a powerful tool in facilitating eHealth adoption among older adults by creating positive perceptions of eHealth, particularly when aided by supportive social networks.

The perceived positive attributes of eHealth, such as ease of use, convenience, and effectiveness, could exert a significant influence on promoting long-term adoption of eHealth among older adults. Social influences, including social learning, can increase the propensity to adopt and continue to use eHealth technologies if they are perceived as easy to learn, user-friendly, accessible, and able to provide meaningful benefits.

## Mooring factors

### Personal and psychological concerns

Multi-level mooring factors were also identified, which could make older adults hesitant to use medical services in a new way outside of the traditional methods. One of the most frequently cited factors hindering the use of eHealth was a concern about security.

*“Because I'm assuming if there's a portal, and maybe I'm wrong about this, but I'm assuming that my Medicare number will be on there. And if it is, then someone else will get it. And we were instructed from 50 years ago, don't ever give anybody that number, your social security number, and I’m sure that would be on there too. So yes, I do hesitate. I'm not a fan of it only because of that.”* (ID1033)

Potential inconvenience of eHealth including exposure to unnecessary information that patients might encounter while using eHealth was pointed out as a factor that discouraged the adoption of eHealth.

*“I haven't registered on the portal, or whatever they call it for the [a health plan], because I don't want to get all their junk emails and I don't want to have a log in and all that kind of stuff.”* (ID 1023)

Unfamiliarity with eHealth—arising from a lack of experience, knowledge, or ICT skills—often resulted in low confidence among older adults when accessing healthcare through technology. This lack of confidence contributed to emotional and psychological barriers, including anxiety and fear of technology failure, which, in turn, led to hesitation in adopting eHealth.

*“[A clinic name removed for confidentiality] has sent me on an email, trying to get me to go the Gateway (Note - a patient portal). You know what I mean? To do this, and I just haven't done it, because I'm not sure if myself on that. So I don't know what I'm scared of but I’m just not brave enough to do it.”* (ID 1031)

The limited capability to utilize technologies, partially due to physical limitations among older adults, turned out to be a factor that increases effort expectancy, which in turn prevents older adults from adopting eHealth. As a respondent stated, *“I do not type well, or keyboard well, or whatever. So, it’s an effort to send my questions to the doctors and things like that. So, I’d rather pick up the phone and call them.”* (ID 1035).

One respondent highlighted the importance of making eHealth easier to use because it would allow more older adults to benefit from it given the fact that health is one of the top priorities among older adults.

*“There are a lot of people in my age group in this community and I would say probably close to 100% of them use email, and probably 80% of them use text messaging. They don’t always understand how they can save a picture that comes through a text message, but they can read the text message and respond to a text message. So, I think if we can make things as simple as sending a text message back and forth, an email back and forth”.* (ID1018)

### Lack of social influence

Respondents who did not actively use eHealth were found to have family or friends who rarely used eHealth. One respondent reported that she did not have any conversation about eHealth with other people nor receive any support for eHealth because *“nobody else in my family really uses it. They can all go out and go to the doctor”* (ID1029). Another respondent said, *“I do not know anybody my age, in my circle and including my friends in their 50s, who do (eHealth)”* (ID1001).

### Limited infrastructure

Accessibility to the internet itself was also pointed out as a factor hindering older adults from using eHealth, which largely relates to financial issues. The lack of affordable equipment and internet access limits the seniors’ ability to take advantage of eHealth services. As one participant noted,

*“I think there's definitely a need for seniors to be able to have access. And in order for that to happen, they need to have access to inexpensive equipment. They need to have access to inexpensive internet because they're all basically living on fixed incomes.”* (ID1016)

The lack of digital devices and limited Internet access in certain residential areas could exacerbate the challenge of accessing eHealth services. For example, in a subsidized senior housing complex, one respondent highlighted the absence of digital devices and the limited availability of the internet as a significant obstacle to eHealth adoption among older adults in her community.

*“There's a few people that have no Internet. I'd say most of the building does not have. There’s a 40-unit apartment building. And I'm trying to decide if even half of the people there have cell phones. Probably at least half but not, certainly not everybody. And they're not all smartphones, the ones that do have cell phones.”* (ID1006)

## Discussion

During the pandemic, many older adults struggled to use eHealth and were less likely to benefit from expanded eHealth in the healthcare service system (24–26). This study explored the experiences and perspectives on eHealth among older adults during the pandemic and identified push, pull, and mooring factors influencing their eHealth adoption. Guided by the UTAUT, this study took a multi-level approach, focusing not only on individual-level factors but also on inter-personal and macro-level factors. We applied four core constructs from UTAUT in our interview guide and data analysis. Given that most previous research tended to focus on individual-level factors, this study contributes to the understanding of how social services and programs reflecting macro-level factors could increase eHealth adoption among older adults.

The COVID-19 pandemic was found to be a strong macro-level push factor that forced older adults to use eHealth. The pandemic influenced eHealth adoption among older adults through two different paths. First, the pandemic changed the healthcare system. By deregulation of payment and regulatory policy, eHealth has significantly expanded since 2020. These changes allowed and encouraged healthcare providers to offer many eHealth options like telehealth visits or patient portals (27, 28). Most participants in our study were first exposed to eHealth after the pandemic. They mentioned that they had to use eHealth because their healthcare providers strongly encouraged them to use it during the pandemic. In this respect, healthcare providers also played a critical role as a macro-level push factor for eHealth adoption among older adults. This finding is consistent with previous research based on a nationally representative sample of older adults. The National Poll on Healthy Aging (NPHA) showed that the proportion of older adults who reported that their healthcare providers offered telehealth visits significantly increased from 14% in 2019 before the pandemic to 62% in June 2020 right after the pandemic (29). This means that the states pulling back funding and bringing back the regulations post-COVID may hurt eHealth adoption and progress achieved. Second, COVID-19 also directly influenced older patients to adopt eHealth. It is known that older adults are at the highest risk of having severe complications and death from COVID-19 (CDC, 2023). During the peak of the pandemic, the CDC recommended that older adults should stay home and avoid social interactions if possible (CDC, 2021). Some of our study participants shared that they had fear and/or anxiety they could be exposed to the virus, and that they did not want to have in-person doctor’s visits.

We identified various pull factors that attracted older adults to use eHealth from all four multilevel categories of the UTAUT. These categories include performance expectancy, effort expectancy, social influence, and facilitating conditions (9). Pull factors for eHealth adoption were not significantly different from the factors that have been demonstrated to be facilitators of other technology use among older adults from the UTAUT framework. First, older adults who understand the benefits of eHealth (i.e., high level of performance expectancy) were satisfied with their eHealth use experience and wanted to continue to use eHealth. Second, older adults who are familiar with ICT (i.e., high level of effort expectancy) felt comfortable using healthcare services through ICT and found more benefits of eHealth. Third, we found that social influence matters in developing a positive perception of eHealth, which led to eHealth adoption among older adults. Older adults’ subjective views on new technology often depend on the perception and attitude of the new technologies by their family and friends (30). Also, older adults are more likely to be willing to try new technology when they feel support or encouragement from family and friends (31). Lastly, our findings showed that environmental-level factors such as Internet access or having friends, neighbors, or service providers (e.g., social workers) available to help anytime in their residential setting could increase eHealth adoption among older adults (44).

Both push and pull factors facilitate older adults’ adoption of eHealth. However, pull factors may exert stronger effects on individuals’ intentions to shift behaviors than push factors. To assess the relative strength of these factors, we examined the intensity and contextual significance of participant statements related to push and pull influences. Our findings indicate that pull factors elicited more positive and immediate behavioral intentions, as evidenced by direct participant quotes and thematic coding analysis. This aligns with previous research indicating that pull factors—such as perceived advantages and enjoyment—are often more powerful in driving the adoption and switching intention toward new technologies (32–34). Unlike push factors, which may provide only temporary motivation—such as those related to the COVID-19 pandemic—pull factors can create sustained engagement. Once the pandemic subsides, there is no guarantee that older adults will continue to use eHealth solely due to previous push factors. In contrast, enhancing older patients’ favorability toward eHealth through pull factors could lead to long-term acceptance. For example, a Medicare claims analysis found that eHealth utilization among older adults declined in late 2021 compared to its peak during the pandemic (35), underscoring the transient nature of push-driven adoption.

We identified multi-level mooring factors that contrasted with the pull factors. For example, older adults who did not perceive significant benefits from eHealth were less likely to use it (i.e., low performance expectancy). Those with limited experience using ICTs in their daily lives or who found technology difficult to navigate often felt overwhelmed when asked to engage with telehealth or patient portals (i.e., low effort expectancy). This lack of understanding of eHealth, combined with unfamiliarity with ICT use, frequently led to psychological barriers such as anxiety or fear of using eHealth—factors that significantly influenced eHealth adoption (36, 47). Additionally, older adults who did not have friends or family using eHealth lacked the social motivation to adopt and maintain its use (i.e., limited social influence). Furthermore, those without personal ICT devices or with restricted internet access faced challenges in effectively utilizing eHealth services (i.e., unsupportive facilitating conditions) (51).

In general, the UTAUT successfully captured most information regarding the facilitators for and barriers to eHealth adoption that we learned from the interviews. However, the UTAUT does not cover some important individual-level factors. First, we found that personalities such as curiosity or self-motivation about the new technology made older adults try the new way for receiving healthcare services. Second, although some study participants possessed all four multi-level pull factors – knowledgeable on eHealth, familiar with ICTs, having friends and family who are using eHealth, and living in the environment supportive to use eHealth-, they did not want to use eHealth just because they strongly prefer in-person doctor visits. Third, physical and/or cognitive limitations could hinder older adults from using eHealth. Lastly, concerns over a perceived lack of control over information through ICT were critical factors for some older adults in their decision to use eHealth. There have been many efforts to extend the UTAUT to capture the comprehensive determinants of various technology use and behavior (14, 62). Our findings show that individuals’ preferences and needs should be added to the UTAUT to explain eHealth adoption among older adults.

One of the limitations of this study is that the findings are based on a small sample that is primarily a convenience sample. Although we tried to include participants from a variety of socioeconomic statuses and different residential settings (e.g., subsidized-senior housing, CCRC, 55+ age restricted community, and regular community), there could be some potential bias from the small size of the convenience sample. Another limitation is that all the interviews were conducted through an online video conference system, Zoom. Although we provided support to set up a zoom meeting if needed, this new interview environment could impact some interviewees who were not familiar with this technology or who did not feel comfortable speaking through ICTs. Lastly, there is the possibility of a novelty effect among participants who adopted eHealth for the first time during the pandemic. The initial excitement or curiosity associated with trying something new could have temporarily increased their willingness to engage in eHealth, thereby potentially inflating positive attitudes and adoption rates. However, as we found from the interviews, older adults often face additional barriers to sustained use—such as limited technology skills, security or privacy concerns, and uncertainty regarding the usefulness of eHealth—factors that can diminish initial enthusiasm over time. Future research could more closely track long-term adoption patterns to distinguish between short-lived novelty-driven uptake and sustained, meaningful engagement in eHealth.

During COVID-19, eHealth has shed light on the opportunities that exist to increase patient access to healthcare through virtual visits with lower costs (35). It has also been demonstrated that eHealth increased patient satisfaction and decreased emergency room use (37). Our findings show the current snapshot of eHealth inequality among the older population. Understanding push, pull, and mooring factors associated with older adults’ intention to adopt eHealth is critical for developing programs that help older adults continue to use eHealth even after the pandemic (38, 56). Also, this study suggests that individuals are influenced by multilevel environments, and their behavior should be understood through interactions between various environments around them (39). In particular, macro-level factors matter for older adults in that individual motivational determents are significantly influenced by social and environmental contexts in this population. This study indicates areas to increase productive use of eHealth within older populations, such as education or support within social networks (55).

A promising area for future research is examining whether the adoption of eHealth during the pandemic has led to lasting behavior change among older adults. Specifically, future studies could explore whether eHealth use has become a habitual part of healthcare routines or if older adults are likely to revert to in-person care as pandemic-related restrictions have eased. Understanding these long-term patterns will be essential for designing interventions that sustain eHealth engagement beyond the temporary push factors created by COVID-19.

## Data Availability

The original contributions presented in the study are included in the article/supplementary material, further inquiries can be directed to the corresponding author.
